# Oral Administration of *Limosilactobacillus reuteri* KBL346 Ameliorates Influenza Virus A/PR8 Infection in Mouse

**DOI:** 10.1007/s12602-024-10301-8

**Published:** 2024-06-29

**Authors:** Doseon Choi, Sung Jae Jang, Sueun Choi, SungJun Park, Woon-Ki Kim, Giljae Lee, Cheonghoon Lee, GwangPyo Ko

**Affiliations:** 1 Department of Environmental Health Sciences, Graduate School of Public Health, Seoul National University, Seoul, Republic of Korea; 2 KoBioLabs, Inc, Seoul, Republic of Korea; 3 N-Bio, Seoul National University, Seoul, Republic of Korea; 4 Institute of Health and Environment, Seoul National University, Seoul, Republic of Korea; 5 Division of Environmental Health Sciences, College of Public Health, The Ohio State University, Columbus, OH USA

**Keywords:** Immunomodulation, Influenza virus, *Limosilactobacillus reuteri* KBL346, Lung, Microbiome

## Abstract

Influenza virus infection is an important public-health concern because of its high transmissibility and potential for severe complications. To mitigate the severity and complications of influenza, probiotics containing *Lactobacillus* are used and generally recognized as safe. We evaluated the anti-influenza effect of *Limosilactobacillus reuteri* (*L. reuteri*) KBL346, isolated from the fecel sample of healthy South Koreans, in mice. BALB/c mice were orally administered live and heat-inactivated *L. reuteri* KBL346. After infection with influenza virus (A/Puerto Rico/8/34) 0.5 times the 50% lethal dose (LD_50_), body weight loss was improved and recovery was accelerated. Furthermore, *L. reuteri* KBL346 improved body weight loss and survival rate of mice infected with 4 times the LD_50_ of influenza virus. Heat-inactivated *L. reuteri* KBL346 reduced the viral titer in the lung and the plasma immunoglobulin G level. Expression levels of genes encoding inflammatory cytokines, such as interferon-γ and toll-like receptor 2 (*Tlr2*), were decreased in the lung tissues of mice administered *L. reuteri* KBL346. Live and heat-inactivated *L. reuteri* KBL346 increased the expression level of *Adamts4*, which promotes recovery after infection, and decreased that of *Tlr2*. The α-diversity of the gut microbiome was modulated by the administration of *L. reuteri* KBL346. In addition, the structure of the gut microbial community differed according to the degree of weight loss. *L. reuteri* KBL346 has the potential to alleviate disease severity and improve histopathological changes in mice infected with influenza A/PR8, suggesting its efficacy as a probiotic against influenza infection.

**Supplementary Information:**

The online version contains supplementary material available at 10.1007/s12602-024-10301-8.

## Introduction

Infectious diseases caused by influenza virus are commonly referred to as influenza or ‘the flu’. Approximately 5–15% of the global population experiences influenza infection annually. Every year, there are three to five million severe cases and up to 650,000 respiratory-related deaths worldwide [1]. International health organizations and research institutes, including the World Health Organization, develop vaccines against seasonal influenza. However, because influenza virus readily mutates, the selection of strains for use in the vaccine is problematic. The worldwide spread of the coronavirus disease 2019 (COVID-19) has increased interest in immunity and health, which has led to an increase in the demand for probiotics.

As living microorganisms, probiotics typically provide health benefits by improving or restoring the intestinal flora. In fact, there is an increasing trend towards scientifically substantiating health-promoting effects of probiotics. Some strains of probiotic bacterial taxa, such as *Lactobacillus* and *Bifidobacterium*, maintain the balance of the gut microbiota and activate the immune system, especially after the elimination of harmful bacteria by antibiotics*. Bifidobacterium lactis* and *Lactobacillus acidophilus* alleviate gas and bloating and prevent constipation. *Lacticaseibacillus rhamnosus* (*L. rhamnosus*) and *Bifidobacterium lactis* prevent allergies and reduce the likelihood of developing asthma. *Saccharomyces boulardii* prevents acute gastrointestinal infections.

In most studies on the effects of probiotics on influenza infection, immune modulation has been emphasized, which can change rapidly following infection [2–14]. However, to our knowledge, effects of orally administered bacteria on the microbiome of influenza-infected mice have not been investigated. Live bacteria can reduce the severity of influenza infection [6, 10, 12, 14, 15]. Influenza can be prevented by inactivated bacteria or bacterial cell-surface polysaccharides, suggesting that not only microbial activity but also microbe-associated molecular patterns trigger immune responses [3, 7–9, 13]. Based on their potentials as functional foods or supplements to alleviate respiratory infections, inactivated bacteria are expected to show similar efficacy but be less costly (*e*.*g*., there is no need to enumerate viable cells) than live bacteria [16]. Depending on species or strains, bacteria can modulate immune responses through a variety of mechanisms. However, there is disagreement over whether immunoactivation or immunosuppression benefits the host during influenza infection.

We reported that *Lactobacillus* isolates from the feces of healthy South Koreans inhibited the replication of murine norovirus in RAW264.7 cells [11]. On this basis, we identified *Limosilactobacillus reuteri* (*L. reuteri*) KBL346 and *L. rhamnosus* KBL352 as candidate probiotics. *L. reuteri* inhibits the growth of intestinal pathogens, maintains integrity of the intestinal barrier, and modulates host immunity [17]. Another research group suggested that oral administration of live *L. reuteri* attenuated allergen-induced asthma [18]. Although they are different anatomic compartments and their relationship is unclear, probiotics in gut can affect the lungs. Indeed, either germ-free or specific pathogen-free (SPF) mice fed antibiotic cocktails have abnormal immune responses to respiratory infections [19–23]. Some strains of *L. rhamnosus* are used as probiotics and are particularly effective against infections of the female urogenital tract, including challenging cases of bacterial vaginosis [24]. *L. rhamnosus* and *L. reuteri* are frequently found in the genitourinary tracts of healthy females and promote the re-establishment of the bacterial balance during active infections caused by dysbiotic overgrowth [25–27]. The mice treated with *L. rhamnosus* M21 also showed less severe pneumonia due to influenza infection [12].

Therefore, in this study, we investigated the anti-influenza effect of *L. reuteri* KBL346 in an in vivo mouse model. We assessed body weight loss and survival rate of mice, changes in markers of immune responses due to influenza infection, and shifts of gut microbial communities as results of the oral-administration of *L. reuteri* KBL346.

## Materials and Methods

### Virus

Influenza PR8 (PR8, H1N1, A/Puerto Rico/8/34), which can infect mice, was obtained from Prof. Nam-Hyuk Cho at Seoul National University College of Medicine. Briefly, the virus seed was inoculated in allantoic fluid of 10–12-day-old embryonated chicken eggs, incubated at 37 °C for 48 h, collected, and enumerated with the plaque assay as described previously [28]. The virus stock was stored at −80 °C until use.

### Bacteria

*L. reuteri* KBL346 (*L. reuteri* KCTC 18428P) and *L. rhamnosus* KBL352 (*L. rhamnosus* KCTC 18427P) were isolated from fresh fecal samples of South Koreans [11]. Both *Lactobacillus* spp. were cultivated on Lactobacilli MRS Agar (BD Difco, Sparks, MD, USA) with 0.05% L-cysteine-hydrochloride at 37 °C overnight under anaerobic conditions. Lyophilized powders of both *Lactobacillus* spp. were prepared by KoBioLabs, Inc (Seoul, Republic of Korea). The viable count of lyophilized *L. reuteri* KBL346 was 1.39 × 10^11^ colony forming units (CFU)/g and that of *L. rhamnosus* KBL352 was 1.51 × 10^11^ CFU/g.

To prepare heat-inactivated *L. reuteri*, 0.125 g of lyophilized powder was washed twice with 3.5 mL of 1 × phosphate-buffered saline (PBS) and then re-suspended in 1× PBS. Bacterial suspensions were heated at 55 °C for 30 min as described previously [4, 29]. Heat-inactivated *L. reuteri* was stored at −20 °C until use. Mice were orally administered bacteria at 1.0 × 10^9^ CFU/200 µL daily from 7 days before infection until the end of the experiment.

### Immunomodulatory Effects of *L. reuteri* On RAW264.7 Cells

The murine macrophage cell line RAW264.7 were cultured in Dulbecco’s modified Eagle medium (DMEM) supplemented with 10% fetal bovine serum and 1% penicillin–streptomycin. To confirm cytokine expression, RAW264.7 cells were seeded onto six-well plates at 5 × 10^5^ cells/well overnight. Next, *L. reuteri* KBL346 was added at densities of 5 × 10^5^, 5 × 10^6^, or 5 × 10^7^ CFU/well and plates were incubated for 24 h at 37 °C in a CO_2_ incubator. After incubation, cells were washed twice with cold 1× PBS and stored at −80 °C.

### Experimental Animals

All experiments involving mice were conducted with protocols approved by the Institutional Animal Care and Use Committee of Seoul National University (SNU-210521–4-1). SPF, 5- or 6-week-old female BALB/c mice were obtained from Raonbio, Inc. (Yongin-si, Republic of Korea). Mice were housed in an animal biosafety level-2 animal facility at College of Pharmacy, Seoul National University. Mice were randomly grouped into five mice per cage and allowed to acclimatize for 2 weeks. Mice were monitored daily for survival and body weight from 1 week before infection until the end of the experiment. The survival rate was evaluated based on death or, as a humane endpoint, loss of 30% of the initial body weight and was calculated using the Kaplan–Meier method [30].

### Lethal Dose

In the primary 50% lethal dose (LD_50_) test, 8-week-old BALB/c mice were divided into six groups (10 mice each). Mice in each group were anesthetized and intranasally administered PR8 virus suspension in PBS at 10^2^, 10^3^, 10^4^, 10^5^, or 10^6^ plaque-forming units (PFU)/mouse.

Based on the calculated primary LD_50_, the final LD_50_ was determined as follows. Eight-week-old BALB/c mice were divided into five groups (fifteen mice per each group). The mice were anesthetized and intranasally administered PR8 virus suspension in PBS at 7,500, 15,000, 30,000, or 60,000 PFU/mouse. Calculation of the primary or final LD_50_ is shown in Tables S1 and S2 [31].

### Infection Model

**Fig. 1:**
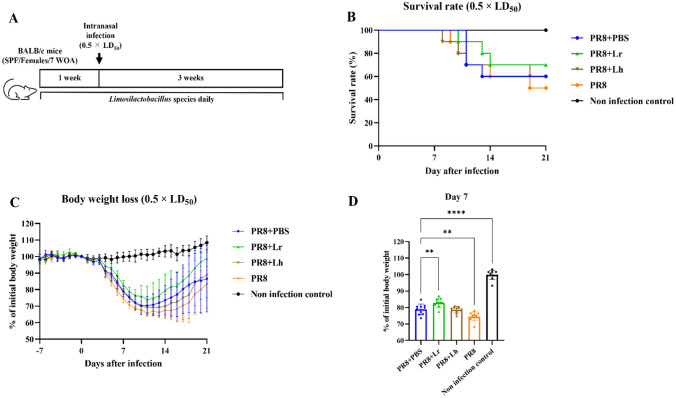
Alleviations of body weight loss and improvement of survival rate by oral administration of *L. reuteri* KBL346 in mice. **A **Experimental design to confirm the effect of *Lactobacillus* on PR8-infected mice (n = 10). **B **Survival rates after infection with 0.5 times the LD_50_ of PR8. The significance of differences in survival was calculated using the log-rank (Mantel–Cox) test. **C **Changes in body weight loss at 21 days after infection. **D **Percentages of body weight at day 7 post-infection. Data in **C **to **D **are the means ± standard deviation. Significance was determined using one-way ANOVA. ** *P* < 0.01, **** *P* < 0.0001

**Fig. 2:**
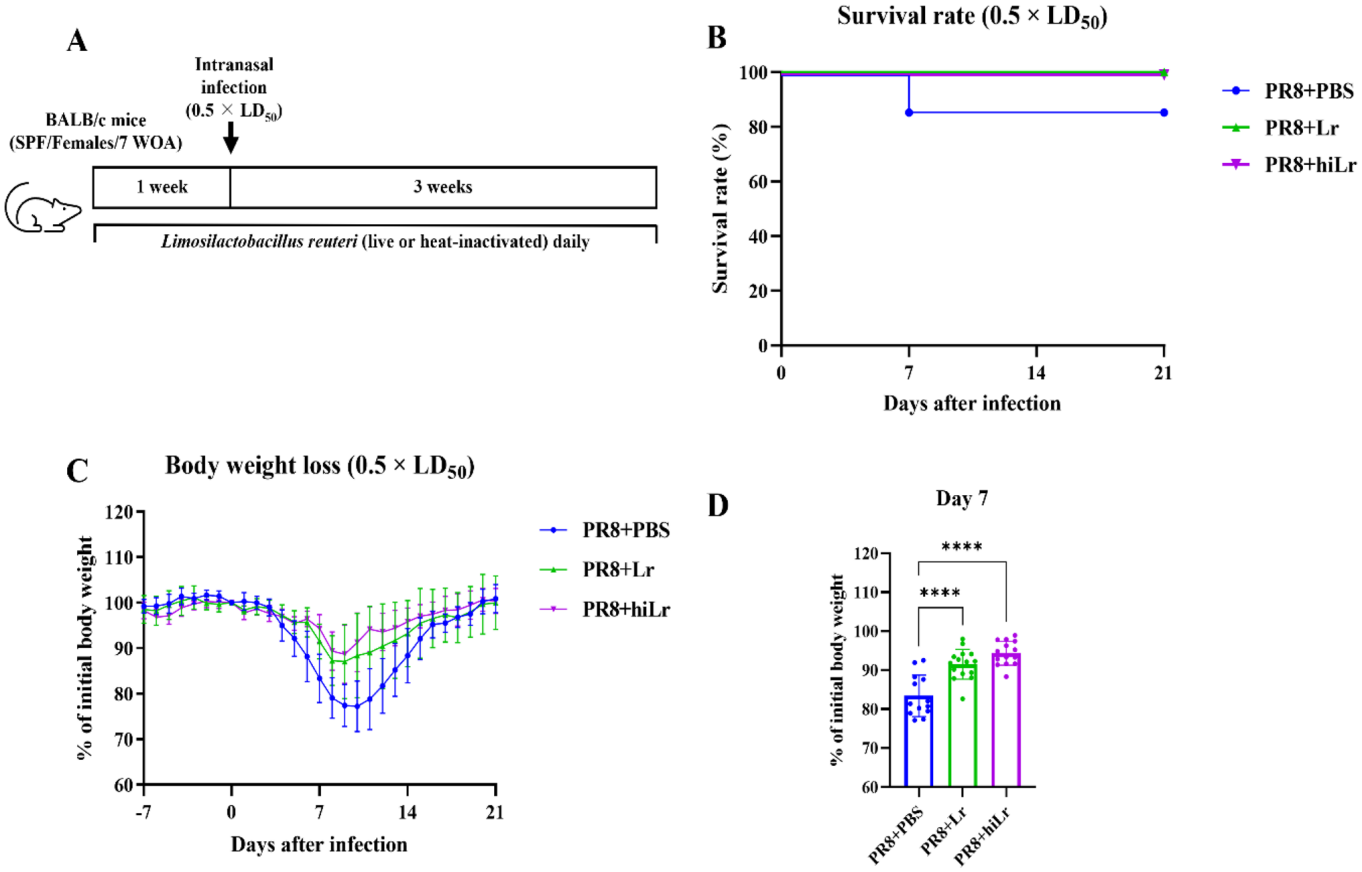
Alleviations of body weight loss and improvement of survival rate by oral administration of heat-inactivated *L. reuteri* KBL346. **A **Experimental design to confirm the effect of heat-inactivated *L. reuteri* KBL346 on PR8-infected mice (n = 15). **B **Survival rate after infection with 0.5 times the LD_50_ of PR8. The significance of differences in survival was calculated using the log-rank (Mantel–Cox) test. **C** Changes in body weight loss at 21 days after infection. **D **Percentages of body weight at day 7 post-infection. Data in **C **to **D **are the means ± standard deviation. Significance was determined using one-way ANOVA. **** *P* < 0.0001

All infection models were performed as described in previous study with some modifications [32]. In the first experiment, 7-week-old BALB/c mice were divided into seven groups (ten mice per each group). Mice were subjected to administration of *L. reuteri* KBL346 (1.0 × 10^9^ CFU/200 µL), *L. rhamnosus* KBL352 (1.0 × 10^9^ CFU/200 µL). Following treatment for 1 week, mice were anesthetized and intranasally administered PR8 virus suspension in PBS (50 µL) with 0.5 times the LD_50_ (LD_50_ = 9.0 × 10^3^ PFU/mouse). For the second to fourth experiments, the *L. rhamnosus *the heat-inactivated *L. reuteri* KBL346 group was added. The dose of heat-inactivated *L. reuteri* KBL346 was 1.0 × 10^9^ CFU/200 µL. From the start to the end of the second to fourth experiments, the administration method was identical to that in the first experiment. In the second and third experiments, mice were infected with influenza virus at 0.5 times the LD_50_, and in the fourth experiment, with influenza virus at 4 times the LD_50_. Details of these experiments are provided in Figs. 1A, 2A, S1A, and 5A.

### Measurement of PR8 Titers in Lung Samples

At 7 days after infection, lung samples were homogenized in 1 mL of PBS. Homogenates were serially diluted in serum-free medium at 4 °C. PR8 virus titers in lung samples were quantified using the plaque assay as described previously with some modifications [33]. Briefly, Madin–Darby canine kidney (MDCK) cells were plated in six-well plates and 0.5 mL of sequentially diluted lung sample was added to the MDCK cell monolayers in duplicate. Next, plates were placed in an incubator at 37 °C for 1 h and washed twice with PBS. We prepared an agar mixture consisting of 1.0% SeaPlaque^™^ Agarose (Lonza, Rockland, ME, USA), DMEM (Life Technologies Corporation, Grand Island, NY, USA), 10 mM 2-[4-(2-hydroxyethyl)piperazin-1-yl]ethanesulfonic acid (HEPES, Life Technologies Corporation), 50 µg/µL penicillin–streptomycin (Life Technologies Corporation), and 2 µg/mL N-tosyl-L-phenylalanine chloromethyl ketone-treated trypsin (Sigma-Aldrich, St. Louis, MO, USA). This agar mixture was added to the wells and allowed to solidify at room temperature for 20 min. Following incubation for 3 days at 37 °C, 2 mL of 70% ethanol were added to deactivate PR8 and preserve MDCK cells. PR8 plaques were visualized using 1% crystal violet solution (Sigma-Aldrich).

### RNA Isolation and Quantitative Real-time PCR

At 7 days after infection, mice (n = 60) were euthanized. Lung tissues were harvested aseptically for RNA extraction using the RNeasy Mini Kit (Qiagen, Hilden, Germany) following the manufacturer’s instructions; 1 µg was used to prepare cDNA with the TOPscript cDNA Synthesis Kit (Enzynomics, Daejeon, Republic of Korea). Quantitative real-time PCR (qRT-PCR) was carried out using the cDNAs, the Power SYBR Green PCR Master Mix (Applied Biosystems, Foster City, CA, USA), and gene-specific primers listed in Table S3. Conditions for qRT-PCR included first step 95 °C for 10 min and followed by 40 cycles of 95 °C 15 s, 60 °C 1 min. The StepOnePlus^™^ Real-time PCR System (Applied Biosystems, Foster City, CA, USA) was used to assess relative gene expression levels, which were calculated using the comparative CT method (2^−ΔΔCt^ method). Glyceraldehyde 3-phosphate dehydrogenase gene (*Gapdh*, NCBI reference sequence NM_008084) was used as the reference gene with the primers suggested in Table S3.

### Measurement of Anti-influenza Virus PR8 Antibody Titer

Recombinant influenza A H1N1 (A/Puerto Rico/8/1934) hemagglutinin and influenza A H1N1 (A/Puerto Rico/8/1934) hemagglutinin-specific mouse monoclonal antibody were purchased from Sino Biological (Beijing, China).

For indirect enzyme-linked immunosorbent assay (ELISA), 1 µg of recombinant hemagglutinin protein was coated onto a 96-well microplate in 100 mM carbonate–bicarbonate buffer (pH 9.6) and blocked with OptEIA Assay Diluent (55,213, BD Biosciences, Franklin Lakes, NJ, USA) for 1 h at room temperature. Following five washes with 1× PBS, plasma from PR8 virus-infected mice was added and incubated for 2 h at room temperature. Unbound antibodies were eliminated by washing five times with 1× PBS containing Tween 20. The signal was developed based on the enzymatic reaction of horseradish peroxidase-conjugated anti-mouse immunoglobulin G (IgG) with 3,3′,5,5′-Tetramethylbenzidine (TMB) substrate (555,214, BD Biosciences). The absorbance at 450 nm was assessed and the background absorbance at 570 nm was measured.

### Analysis of Lung Histopathology

After euthanizing mice at day 7 post-infection, lungs were fixed in 10% neutral buffered formalin. Then, lungs were embedded in paraffin wax, sectioned at 4-µm thickness, and stained with hematoxylin and eosin (H&E). Histopathological images were digitally scanned by T&P BIO (Gwangju, Republic of Korea). Micrographs (10 ×) were obtained using a digital scanner (Panoramic SCAN II, 3DHistech Ltd., Budapest, Hungary).

### Metagenome Sequencing and Analysis

Fecal samples were collected from PR8-infected mice and DNA was extracted using the QIAamp FAST DNA Stool Mini Kit (Qiagen GmbH, Hilden, Germany) according to the manufacturer’s instructions. The V4–5 hypervariable region of 16S rRNA gene was amplified and barcoded by PCR using the Premix Taq^™^ (TaKaRa Bio Inc., Shiga, Japan) and the primers 515F and 926R, which have barcode adaptors for sequencing. Samples containing 100 ng of DNA were purified using the QIAquick PCR Purification Kit (Qiagen GmbH, Hilden, Germany). DNA was quantified using the Quant-iT^™^ PicoGreen^™^ dsDNA Assay Kit (Thermo Fisher Scientific, Waltham, MA, USA).

Pooled PCR amplicons (5 µL each) were sequenced using the MiSeq Reagent Kit ver. 3 (600 cycles) on the MiSeq platform (Illumina Inc., Illumina Way, CA, USA). For quality filtering and downstream analysis of α-diversity, β-diversity, and composition, we used the Quantitative Insights into Microbial Ecology 2 (QIIME2) software package (version qiime2-2021.2; https://qiime2.org) with standard protocols. Using DADA2, sequences were denoised, resulting in amplicon sequence variants. The RDP classifier in QIIME2 was employed to assign taxa with the Greengenes database ver. 13.8. β-diversity, representing the diversity partitioning among communities, was computed using the weighted UniFrac distance metric. To assess the statistical significance of differential sample clustering on principal coordinates analysis (PCoA) plots, we performed permutational multivariate analysis of variance (PERMANOVA) with 999 permutations.

### Statistical Analysis

Data are expressed as means ± standard deviation of the mean of independent experiments. When appropriate, data were analyzed using the Mann–Whitney U-test or one-way ANOVA followed by Dunnett’s post hoc multiple comparison test in Prism ver. 9.01 software (GraphPad Software, Inc., La Jolla, CA, USA). Survival analysis was conducted with the log-rank (Mantel–Cox) test in Prism.

## Results

### Oral Administration of *L. reuteri* KBL346 Alleviates Body Weight Loss and Improves Survival in Influenza-infected Mice

We investigated the effect of oral administration of *L. reuteri* KBL346 on influenza infection in mice (Fig. 1A). Body weight loss was alleviated and survival rate was increased in mice administered *L. reuteri* KBL346 compared to mice with 1× PBS, as the negative control group, or mice with *L. rhamnosus* KBL352 (Fig. 1B and C). Body weight loss of mice administered *L. reuteri* KBL346 was significantly lower compared to the control group or *L. rhamnosus* KBL352 at day 7 post-infection (Fig. 1D).

### Heat-inactivated and Live *L. reuteri* KBL346 Have Comparable Efficacies

We investigated the effect of *L. reuteri* KBL346 on PR8 infection in mice (Fig. 2A). *L. reuteri* KBL346 did not showed statistically significances on improvements of survival rate with 0.5 times the LD_50_ of PR8 (Fig. 2B). Mice administered heat-inactivated *L. reuteri* KBL346 had similar body weight loss to those administered live bacteria (Fig. 2C and D).

### Heat-inactivated *L. reuteri* KBL346 Reduces PR8 Titer in the Lungs

We assessed body weight loss and the PR8 virus titer in the lungs of mice with heat-inactivate *L. reuteri* KBL346 (Fig. S1). Compared to the control group, mice with heat-inactivated *L. reuteri* KBL346 showed significant reductions of the lung PR8 virus titer (Fig. S1D).

### Heat-inactivated *L. reuteri* KBL346 Modulates the Inflammatory Response in the Plasma and Lungs Following PR8 Virus Infection

**Fig. 3:**
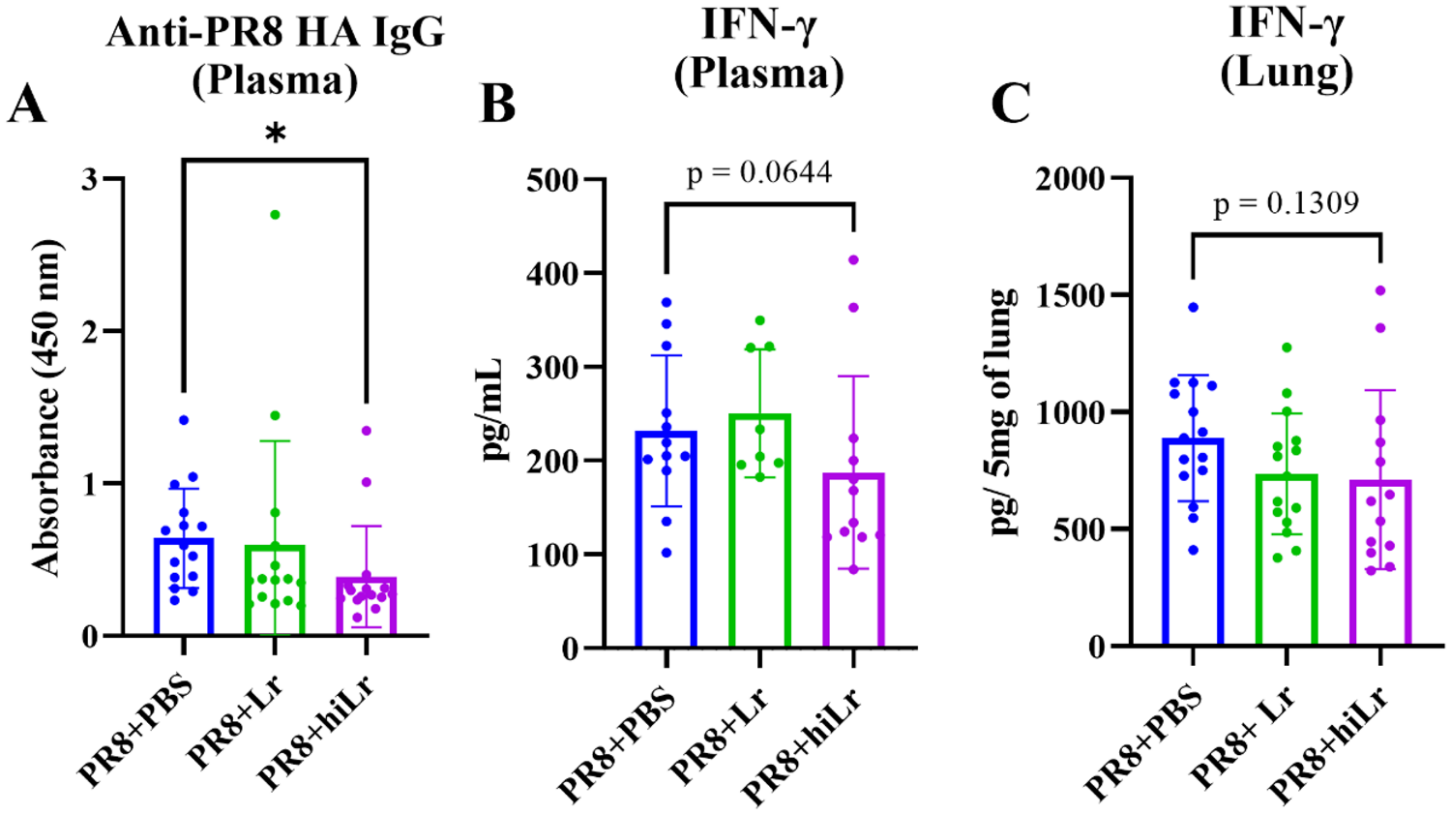
Changes in inflammatory responses in plasma and lungs of mice infected with PR8 by oral administration of heat-inactivated *L. reuteri* KBL346. **A **Plasma anti-PR8 hemagglutinin IgG level after administration of live or heat-inactivated *L. reuteri* KBL346 (n = 15). **B **Plasma IFN-γ level after administration of live or heat-inactivated *L. reuteri* KBL346 (n = 8–12). **C **Lung IFN-γ level after administration of live or heat-inactivated *L. reuteri* KBL346 (n = 13–15). Data are the means ± standard deviation. Significance was determined using one-way ANOVA. * *P* < 0.05

To determine the effect of heat-inactivated *L. reuteri* KBL346 on inflammatory responses, we assessed interferon (IFN)-γ and IgG levels at day 7 post-infection (Fig. 3A–C). Oral administration of heat-inactivated *L. reuteri* KBL346 reduced the anti-PR8 hemagglutinin IgG level significantly compared to the control group (Fig. 3A). The IFN-γ level was not significantly decreased by the administration of heat-inactivated *L. reuteri* KBL346 (Fig. 3B and C).

### Live and Heat-inactivated *L. reuteri* KBL346 Modulate Immune Responses in the Lungs Following PR8 Virus Infection

**Fig. 4:**
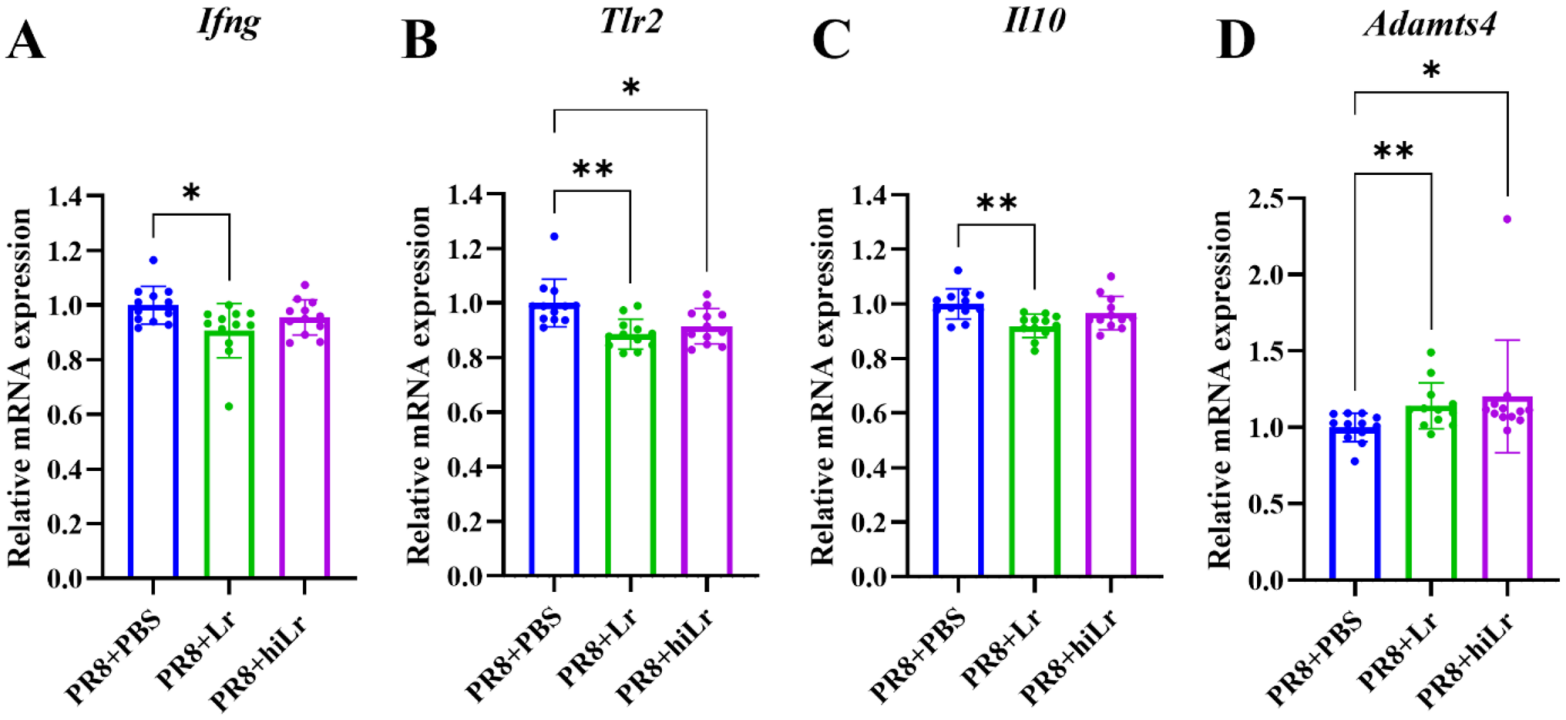
Modulation of immune responses in the lungs of PR8-infected mice after administration of *L. reuteri* KBL346. Expression levels of **A ***Ifng*, **B ***Tlr2*, **C ***Il10*, and **D ***Adamts4* in the lungs of mice administered live or heat-inactivated *L. reuteri* KBL346 (n = 12). Data are the means ± standard deviation. Significance was determined using one-way ANOVA. * *P* < 0.05, ** *P* < 0.01

We assessed expression levels of cytokine-encoding genes in lungs at day 7 post-infection (Fig. 4A–D). Oral administration of *L. reuteri* KBL346 downregulated genes for IFN-γ (*Ifng*), toll-like receptor 2 (*Tlr2*), and interleukin (IL)-10 (*Il10*) compared with the control group (Fig. 4A–C). Heat-inactivated *L. reuteri* KBL346 did not affect the expression levels of *Ifng* and *Il10* in the lungs (Fig. 4A and C) but reduced that of *Tlr2* (Fig. 4B). Live and heat-inactivated *L. reuteri* KBL346 significantly increased the expression level of a disintegrin and metalloproteinase with thrombospondin motifs 4 (*Adamts4*), a damage-responsive fibroblast (DRFib) signature gene (Fig. 4B).

### Interaction of *L. reuteri* KBL346 with RAW264.7 Cells Induces the Expression of Proinflammatory Genes

To evaluate the effect of live *L. reuteri* KBL346 on inflammatory responses, we assessed gene expression levels in RAW264.7 cells (Fig. S2). *L. reuteri* KBL346 showed the upregulation of genes for IL-1β (*Il1b*), IL-6 (*Il6*), tumor necrosis factor (TNF)* (Tnf*), and IFN-β1 (*Ifnb1*) compared with the control (Fig. S2).

### Live and Heat-inactivated *L. reuteri* KBL346 Improve Lung Histopathological Changes

Mice treated with live and heat-inactivated *L. reuteri* KBL346 exhibited fewer histopathological symptoms in the lungs, including reduced bronchial epithelium rupture and necrosis, and atelectasis, compared to the control group (Fig. S3).

### Live and Heat-inactivated *L. reuteri* KBL346 Increase Survival Rate in a 4 times the LD_50_ Infection

**Fig. 5:**
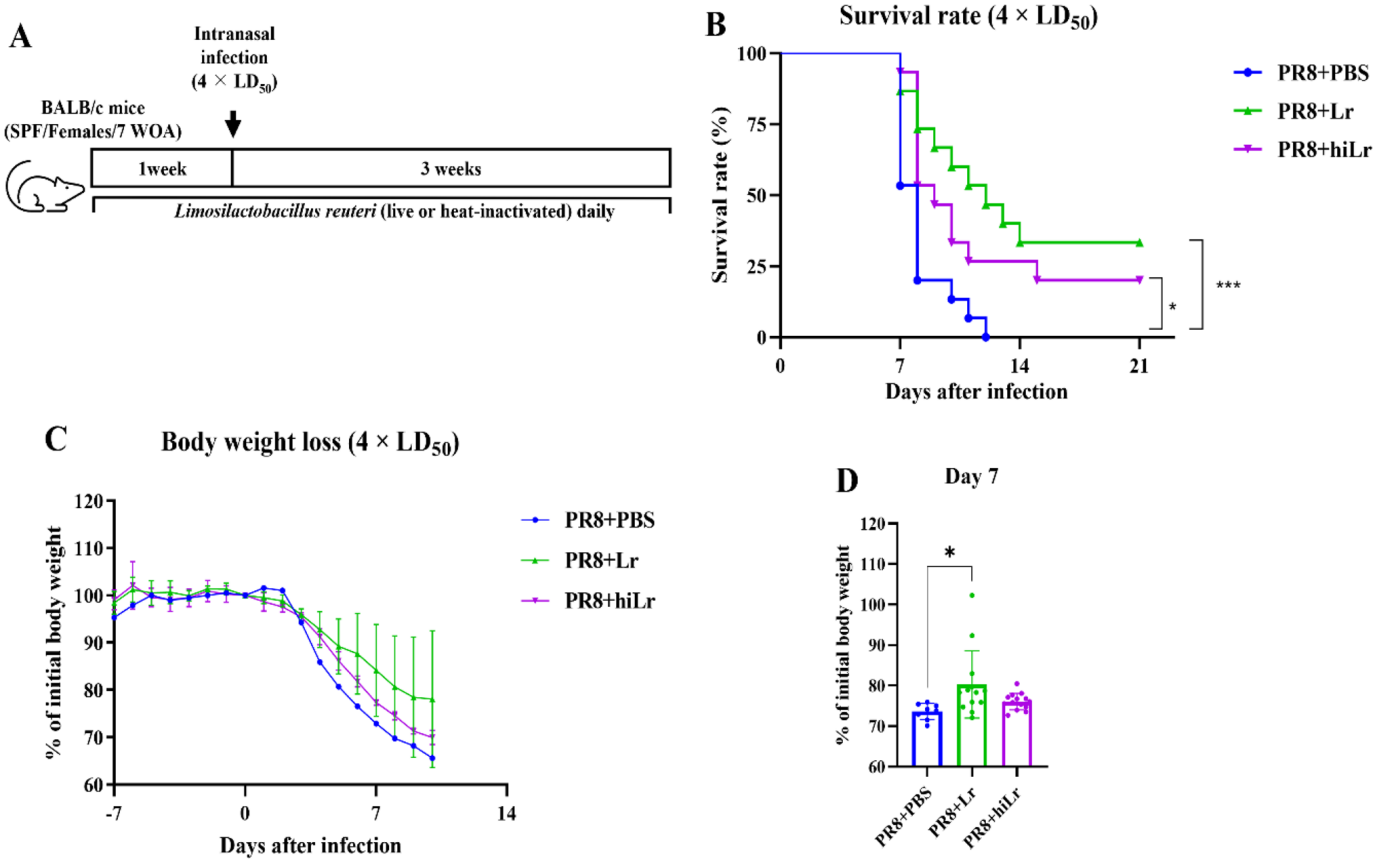
Effects of orally administered *L. reuteri* KBL346 on the survival rate of mice infected with 4 times the LD_50_ PR8. **A **Experimental design to confirm the effect of heat-inactivated *L. reuteri* KBL346 on PR8-infected mice (n = 15). **B **Survival rate after infection with 4 times the LD_50_ of PR8. The significance of differences in survival was calculated using the log-rank (Mantel–Cox) test. **C** Changes in body weight loss at 21 days after infection. **D **Percentages of body weight at day 7 post-infection. Data in **C **to **D **are the means ± standard deviation. Significance was determined using one-way ANOVA. * *P* < 0.05

We evaluated the effects of live and heat-inactivated *L. reuteri* KBL346 at 4 times the LD_50_ of PR8 (Fig. 5A). The survival rate was increased by the oral administration of live or heat-inactivated *L. reuteri* KBL346 (Fig. 5B) Also, live and inactivated *L. reuteri* KBL346 tended to alleviate body weight loss compared to the control group (Fig. 5C and D).

### Changes in Gut Microbial Community Structure with Influenza Infection  

We assessed the microbial diversity in feces. Shannon diversity indices increased significantly after influenza infection (Fig. S4A). The observed operational taxonomic units (OTUs) significantly increased after influenza infection (Fig. S4B). A PCoA based on weighted UniFrac metrics revealed altered patterns and increased distances after influenza infection (Fig. S4C and D).

### Live and Heat-inactivated *L. reuteri* KBL346 Alter Gut Microbial Composition At the Genus Level

Shannon diversity and observed OTU indices of all the groups increased after treatment (Fig. S5A and B). Weighted UniFrac PCoAs and distances showed no significant differences between the control and treatment groups (Fig. S5C and D). The dominant microbial genus was Muribaculaceae_unclassified, whose abundance decreased over time, whereas those of Oscillospira and Clostridiales_unclassified increased (Fig. S5E). On day 6 after infection, the relative abundance of Clostridiales_unclassified was significantly higher in the PBS group compared to the heat-inactivated *L. reuteri* KBL346 group. On the day of infection, the control group had a significantly higher proportion of RF39_unclassified than the live *L. reuteri* KBL346 group (Fig. S5F).

### Food Alters the Gut Microbial Community Structure

**Fig. 6:**
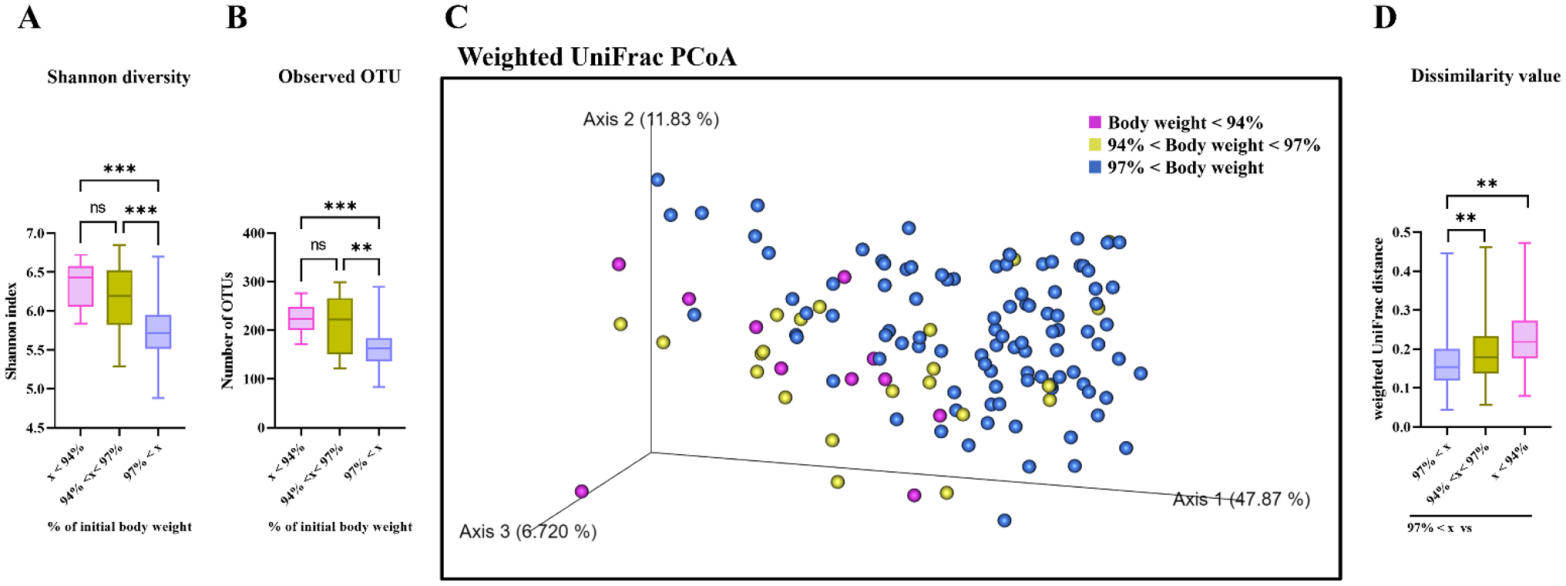
Shifts in gut microbial community structure according to the change in body weight. Analysis of the microbiome of fecal samples (n = 11 for the group with < 94% of the initial body weight; n = 22 for the group with > 94% and < 97% of the initial body weight and n = 98 for the group with > 97% of the initial body weight). **A **Shannon diversity indices and **B **observed OTU indices of the fecal microbiome at 10,885 sequences per sample. Significance was determined using one-way ANOVA. **C **PCoA plot of fecal microbiota structure based on weighted UniFrac metrics. **D **Pairwise weighted UniFrac distances to the group with > 97% of the initial body weight. PERMANOVA was applied (n = 11 permutations for the group with < 94% of the initial body weight, n = 22 permutations for the group with > 94% and < 97% of the initial body weight and n = 98 permutations for the group with > 97% of the initial body weight). Boxplots show the medians with interquartile ranges; whiskers extend from minima to maxima. Differences in microbial composition were evaluated using PERMANOVA based on weighted UniFrac distances. ** *P* < 0.01, *** *P* < 0.001

All mice were grouped according to the degree of body weight loss. Shannon diversity and observed OTU indices significantly increased in the group with < 94% of the initial body weight compared to the group with > 97% of the initial body weight (Fig. 6A and B). PCoA using weighted UniFrac metrics indicated stronger clustering of bacterial communities in the group with > 97% of the initial body weight. Weighted UniFrac distances increased in the group with < 94% of the initial body weight, and in the group with > 94% and < 97% of the initial body weight (Fig. 6C and D).

## Discussion

We evaluated the effect of *L. reuteri* KBL346 on immunity and disease severity following influenza virus infection. In this study, IgG levels in plasma and IFN-γ levels in lungs and plasma of influenza-infected mice were suppressed by heat-inactivated *L. reuteri* KBL346, which corresponded to a reduced PR8 titer. Therefore, *L. reuteri* KBL346 may block the spread of PR8 virus. Also, *Tlr2* was downregulated by live *L. reuteri* KBL346, suggesting suppression of an excessive innate immune response. Indeed, activation of TLR2 by palmitic acid reportedly induces NLR family pyrin domain containing 3 (NLRP3)-mediated IL-1β production in human monocytes [34].

The ability of *L. reuteri* KBL346 to induce *Il1b*, *Il6*, *Tnf*, and *Ifnb1* expression in RAW264.7 cells in vitro suggests protection against influenza infection (Fig. S1). Previous studies have been reported that various probiotics have immunostimulatory effects on macrophages [35–37]. TNF-α and IFN-β, key proinflammatory cytokines, are produced primarily by macrophages [38–40]. TNF-α, which activates nuclear factor-κB, stimulates TNF secretion and upregulates IL-1β and IL-6 [41, 42]. TNF-α acts synergistically with other cytokines to trigger IFN-γ production by T or natural killer cells, and IFN-γ plays a crucial role in infection control until activated cluster of differentiation 8 (CD8) T cells begin to release IFN-γ [43]. In this study, the level of IFN-γ was lowest in the *L. reuteri* KBL346 group, indicating that *L. reuteri* KBL346 attenuates the need for transition to T-cell adaptive immunity. Therefore, *L. reuteri* KBL346 has positive immunomodulatory effects on the immune system of host, including the activation of macrophage.

Oral administration of viable bacteria, such as the butyrate-producing *Clostridium butyricum*, can produce ω-3 fatty acid 18-hydroxy eicosapentaenoic acid. This compound enhances viral clearance by upregulating IFN-λ [44]. Bacteria killed or inactivated by heat have been reported to have similar infection severity reduction effects [3, 7–9]. An effect of heat-inactivated probiotics on influenza virus infection is indirectly supported by increased type I IFN production in mice [3, 8]. In this study, heat-inactivated *L. reuteri* KBL346 reduced histopathological symptoms and viral titers in the lungs during influenza virus infection. Our findings indicate that both live and heat-inactivated *L. reuteri* contributed to a decrease in disease severity following influenza virus infection. These suggest that mechanisms by which *L. reuteri* suppresses influenza virus infection are linked to its cellular constituents, rather than metabolites or activation of viable *L. reuteri* in the intestine. In this study, body-weight deviation of the heat-inactivated *L. reuteri* group was lower than that of the live *L. reuteri* group. Mice treated with heat-inactivated *L. reuteri* showed a high survival rate and an enhanced immune response in the lung after infection with 4 × LD_50_ of PR8. Also, pulmonary immune responses were modulated to a greater degree by live than by heat-inactivated *L. reuteri.* Heat-killed probiotics still maintain strong immunomodulatory effects, making them suitable for oral administration without risks associated with translocation to the bloodstream in vulnerable patients or transfer of antibiotic resistance genes [45, 46]. In addition, their standardization and storage are straightforward [45]. However, live probiotics have unique beneficial effects for host, such as production of anti-microorganism substances and inhibition of pathogen adhesion or colonization. Further studies are necessary to elucidate major effector molecules of *L. reuteri* KBL346, which would enhance its utility as a valuable live or heat-killed probiotic strain for preventing or treating influenza infection.

Although the microbiome changes did not align closely with phylogeny, *L. reuteri* KBL346 has the potential to reshape immunity. Consistent with our findings (Fig. S5F), the relative abundance of RF39_unclassified was decreased during disease induction and after probiotic administration. RF39_unclassified was positively associated with fecal and colonic mucosal samples from dextran sulfate sodium-administered mice [47]. Feeding a mixture of three *Bacillus subtilis* strains reduced the abundance of RF39_unclassified in the cecum, increasing the infection rate of birds orally challenged with *Salmonella enterica* serovar Heidelberg [48]. In a mouse model of multiple sclerosis, the abundance of RF39_unclassified was elevated [49]. Moreover, our finding suggested that clear alterations of gut microbiota occurred in mice with influenza infection due to *L. reuteri* KBL346. Respiratory viral infection can alter the composition of intestinal microbiota and occur microbial dysbiosis in gut and respiratory tract and alterations in the host immune system including activities of immune cells [50, 51]. Especially, influenza infection can affect nutrient usage preferences and antibiotics resistances of cecal microbiota of mice [52]. PCoA plots showed distinct clustering between the groups with > 97% and < 97% of the initial body weight. These suggest that variations in intake quantity can affect the gut microbiome and the host immune system [53, 54]. In mice with severe influenza infection, eating is hampered, perturbing the gut microbiome [54, 55]. Indeed, the abundance of Bacteroidetes increased and those of Muribaculaceae_unclassified and Firmicutes decreased in fasted mice, similar to the finding that the microbiome is altered by the decreased appetite caused by respiratory syncytial virus infection [54]. In other words, inappetence caused by influenza infection leads to changes in the composition of the gut microbiome. However, this study confirmed alterations in gut microbial communities on influenza-infected mice following oral administration of *L. reuteri* KBL346. Therefore, further research is needed to investigate anti-influenza effects and mechanisms of altered gut microbiome due to live or heat-killed *L. reuteri* KBL346 for enhancing our understanding.

Our findings suggest that *L. reuteri* KBL346 modulates *Adamts4* expression, related to enhancement of post-infection recovery. *Adamts4* is expressed in DRFibs [56]. DRFibs are related to the pathways of tissue-damage responses [56]. DRFibs were enriched and remained actively from infection to recovery [56]. However, excessive fibroblast activities increased immune cell infiltration and alveolar inflammation, leading to extensive damages to lung tissues [56]. Our findings of upregulation of *Adamts4* in the live and heat-inactivated *L. reuteri* KBL346 groups indicate reduction of disease severity. A balanced immune response is important for survival with minimal damage. In acute respiratory distress syndrome [57], acute respiratory infection causes collapse and necrosis of the bronchial epithelium, leading to loss of function in the absence of normal gas exchange, *i*.*e*., atelectasis [58]. Further studies using gnotobiotic or transgenic mice are needed to elucidate mechanisms of *L. reuteri* KBL346 for influenza infection.

Other probiotic bacterial taxa that have demonstrated efficacy in mouse models, such as *Bifidobacterium bifidum*, *Bifidobacterium breve*, *Bifidobacterium longum*, *L. rhamnosus*, and *Lactiplantibacillus plantarum*, may enable the prediction and improvement of the health status of mice [2, 3, 5, 6, 10, 12–14]. Moreover, further studies with various factors, including sex disparity [32] and period, should be performed to elucidate the anti-influenza effects of live and heat-inactivated *L. reuteri* KBL346 fully.

## Conclusion

In summary, immune responses in mice were modulated by live and heat-inactivated *L. reuteri* KBL346. Our findings suggest mechanisms for the effect of influenza infection on the gut microbial composition and the crosstalk between *L. reuteri* KBL346 and the immune system. Orally administered KBL346 alleviated severity of influenza infection in mice, indicating *L. reuteri* KBL346 could be used as a probiotic against influenza infection.

## Supplementary Information

Below is the link to the electronic supplementary material.

Supplementary file1 (DOCX 2695 KB)

## Data Availability

The corresponding authors can provide the data upon reasonable request.
