# Supplementary material for: Oral Administration of Limosilactobacillus reuteri KBL346 Ameliorates Influenza Virus A/PR8 Infection in Mouse
Source: Probiotics Antimicrob Proteins. 2024 Jun 29;17(5):3546–58. doi: 10.1007/s12602-024-10301-8 (PMC12532686; doi:10.1007/s12602-024-10301-8)
Supplement: Supplementary file 1 — Supplementary file1 (DOCX 2695 KB) [file 12602_2024_10301_MOESM1_ESM.docx]

**Supplementary Information**

**Manuscript title:** Oral administration of *Limosilactobacillus reuteri* KBL346 ameliorates influenza virus A/PR8 infection in mouse

**Authors:**

Doseon Choi ^1^, Sung Jae Jang ^1,2^, Sueun Choi ^1^, SungJun Park ^1,2,3^, Woon-Ki Kim ^1,4^, Giljae Lee ^1,4^, Cheonghoon Lee ^1,4,5,*^, GwangPyo Ko ^1,2,3,4,*^

^1^Department of Environmental Health Sciences, Graduate School of Public Health, Seoul National University, Seoul, Republic of Korea; ^2^KoBioLabs, Inc, Seoul, Republic of Korea; ^3^N-Bio, Seoul National University, Seoul, Republic of Korea; ^4^ Institute of Health and Environment, Seoul National University, Seoul, Republic of Korea; ^5^ Division of Environmental Health Sciences, College of Public Health, The Ohio State University, Columbus, OH, USA

*Corresponding author email address: shota2@snu.ac.kr (Cheonghoon Lee)

*Corresponding author email address: gko@snu.ac.kr (GwangPyo Ko)

**Number of pages**: 15

**Number of tables:** 3

**Number of figures:** 5

**Table S1** Calculation of the primary LD_50_ of PR8 using the Reed and Muench method

| Log10 virus dilution | Mice | | Cumulative total | | | Percent mortality |
| --- | --- | --- | --- | --- | --- | --- |
|  | Died | Survived | Died | Survived | Total |  |
| -2.70 | 5 | 0 | 11 | 0 | 11 | 11/11 × 100 = 100 |
| -3.70 | 5 | 0 | 6 | 0 | 6 | 6/6 × 100 = 100 |
| -4.70 | 1 | 4 | 1 | 4 | 5 | 1/5 × 100 = 20 |
| -5.70 | 0 | 5 | 0 | 9 | 9 | 0/9 × 100 = 0 |
| -6.70 | 0 | 5 | 0 | 14 | 14 | 0/14 × 100 = 0 |
| -∞ | 0 | 5 | 0 | 19 | 19 | 0/19 × 100 = 0 |

Difference of logarithms = (100-50)/(100-20) = 0.625; end point dilution = -3.70 - (0.625 × 1) = -4.325; 50% end point dilution = 10^-4.325^; the titer of the virus = 10^4.325^ LD_50_/mL

**Table S2** Calculation of the final LD_50_ of PR8 using the Reed and Muench method

| Log10 virus dilution | Mice | | Cumulative total | | | Percent mortality |
| --- | --- | --- | --- | --- | --- | --- |
|  | Died | Survived | Died | Survived | Total |  |
| -3.92 | 10 | 0 | 33 | 0 | 33 | 33/33 × 100 = 100 |
| -4.22 | 10 | 0 | 23 | 0 | 23 | 23/23 × 100 = 100 |
| -4.52 | 9 | 1 | 13 | 1 | 14 | 13/14 × 100 = 92.9 |
| -4.82 | 4 | 6 | 4 | 7 | 11 | 4/11 × 100 = 36.4 |
| -∞ | 0 | 10 | 0 | 17 | 17 | 0/17 × 100 = 0 |

Difference of logarithms = (92.9-50)/(92.9-36.4) = 0.759; end point dilution = -4.52 - (0.759 × 0.301) = -4.748; 50% end point dilution = 10^-4.748^; the titer of the virus = 10^4.748^ LD_50_/mL

**Table S3** Sequences of the primers used for PCR

| Gene | Sequence (5' → 3') | Reference |
| --- | --- | --- |
| *Gapdh* | Forward: 5'-AGGTCGGTGTGAACGGATTTG-3′ | [1] |
|  | Reverse: 5'-TGTAGACCATGTAGTTGAGGTCA-3′ |  |
| *Ifng* | Forward: 5'-TCAAGTGGCATAGATGTGGAAGAA-3′ | [2] |
|  | Reverse: 5'-TGGCTCTGCAGGATTTTCATG-3′ |  |
| *Il10* | Forward: 5'-GCTCTTACTGACTGGCATGAG-3′ |  |
|  | Reverse: 5'-CGCAGCTCTAGGAGCATGTG-3′ |  |
| *Tlr2* | Forward: 5'-TGAAGTCAGCTCACCGATGAA-3′ | [3] |
|  | Reverse: 5'-GTGAGAACCGAGCCTCGGA-3′ |  |
| *Adamts4* | Forward: 5'- TGGCCTCAATCCATCCCAG-3′ | [4] |
|  | Reverse: 5'-AAGCAGGGTTGGAATCTTTGC-3′ |  |
| *Hprt* | Forward: 5'-TTATGGACAGGACTGAAAGAC-3′ | [2] |
|  | Reverse: 5'-GCTTTAATGTAATCCAGCAGGT-3′ |  |
| *Il1b* | Forward: 5'-GTCGCTCAGGGTCACAAGAA-3′ | [5] |
|  | Reverse: 5'-CCACACGTTGACAGCTAGGT-3′ |  |
| *Il6* | Forward: 5'-GCCTTCTTGGGACTGATGCT-3′ | [6] |
|  | Reverse: 5'-AGCCTCCGACTTGTGAAGTG-3′ |  |
| *Tnf* | Forward: 5'-CAGACCCTCACACTCAGATCATCT-3′ | [7] |
|  | Reverse: 5'-CCTCCACTTGGTGGTTTGCTA-3′ |  |
| *fna1b* | Forward: 5'-CAGCTCCAAGAAAGGACGAAC-3′ | [8] |
|  | Reverse: 5'-GGCAGTGTAACTCTTCTGCAT-3′ |  |


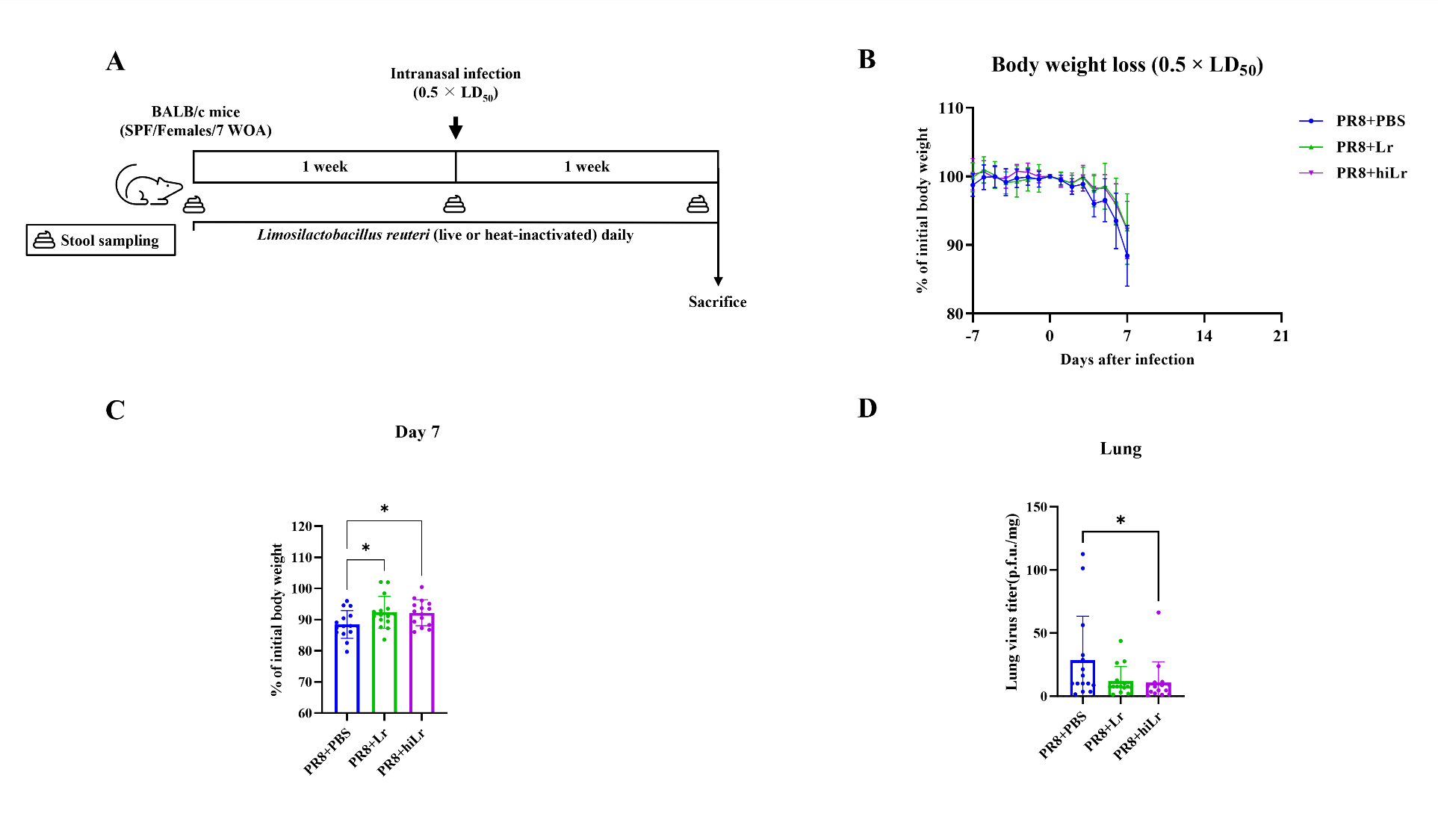


**Fig. S1** Effects of oral administration of heat-inactivated *L. reuteri* KBL346 in PR8-infected mice. (A) Experimental design to confirm the effect of live or heat-inactivated *L. reuteri* KBL346 on lung PR8 titer (n = 15). (B and C) Body weight loss. (D) Mean lung PR8 titer. Data are the means ± standard deviation. Significance was determined using one-way ANOVA. * *P* < 0.05


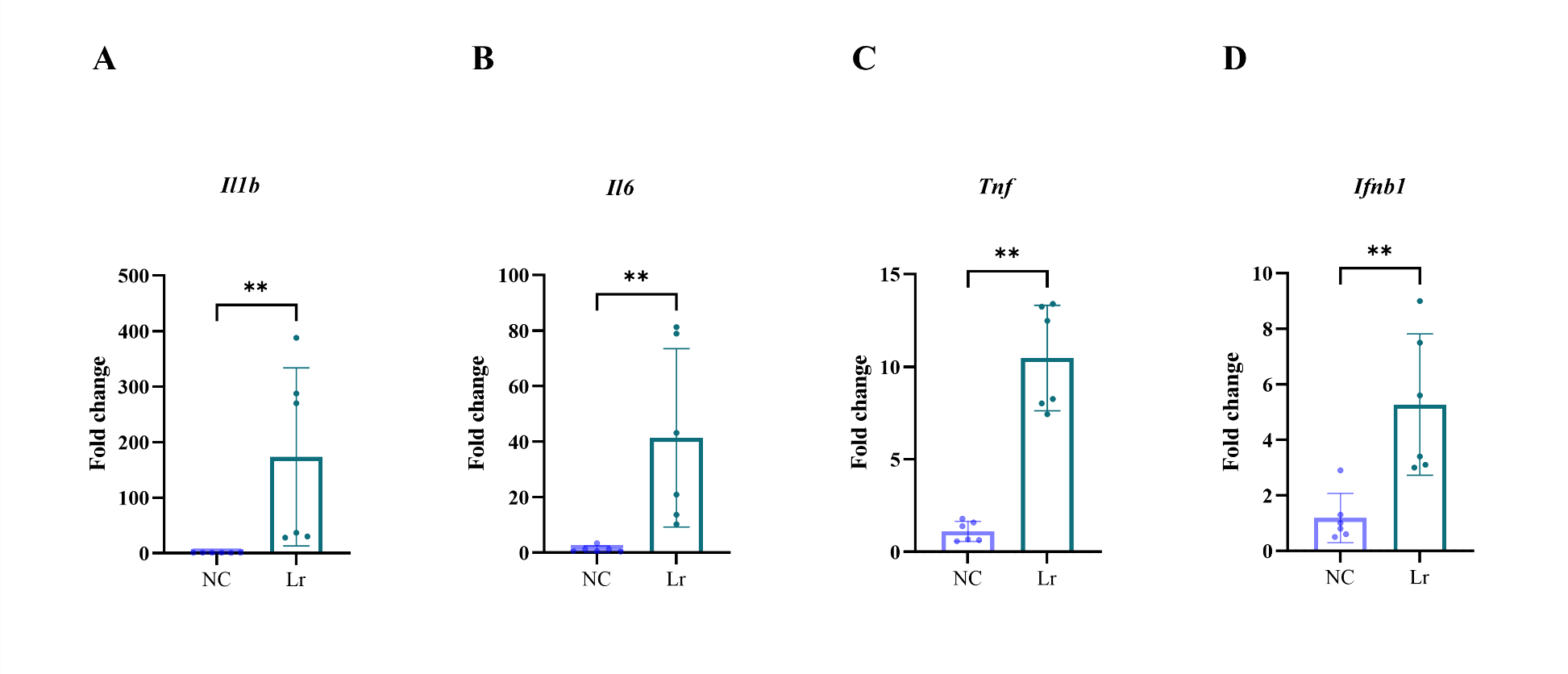


**Fig. S2** Expressions of proinflammatory genes in RAW264.7 cells induced by *L. reuteri* KBL346. Expression levels of (A) *Il1b*, (B) *Il6*, (C) *Tnf*, and (D) *Ifnb1* with live *L. reuteri* KBL346 treatment (n = 6). Data are the means ± standard deviation. Statistical analysis was performed using the Mann–Whitney U-test compared to the negative control. ** *P* < 0.01


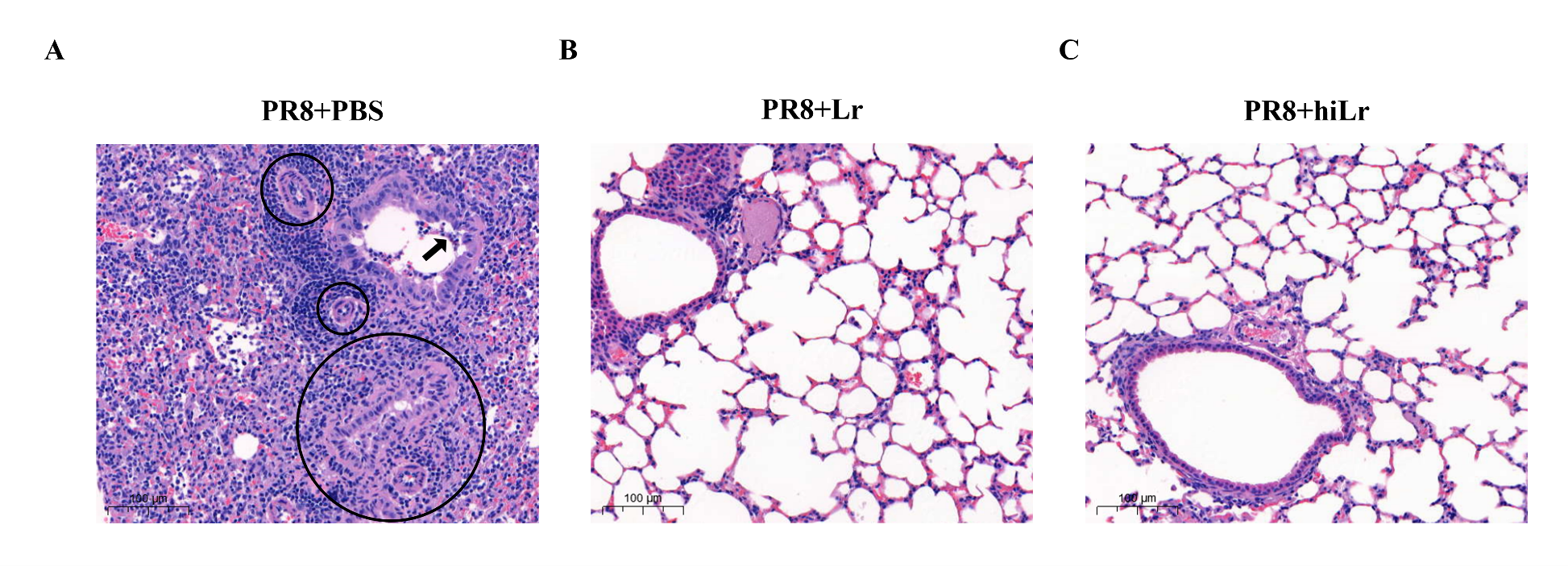


**Fig. S3** Amelioration of lung histopathological changes in PR8-infected mice by *L. reuteri* KBL346. (A–C) Representative H&E-stained images of lung tissues of PR8-infected mice treated with (A) PBS (negative control), and (B) live and (C) heat-inactivated *L. reuteri* KBL346. Arrow: bronchial epithelium rupture and necrosis; Circles: atelectasis; Scale bar: 100 µm


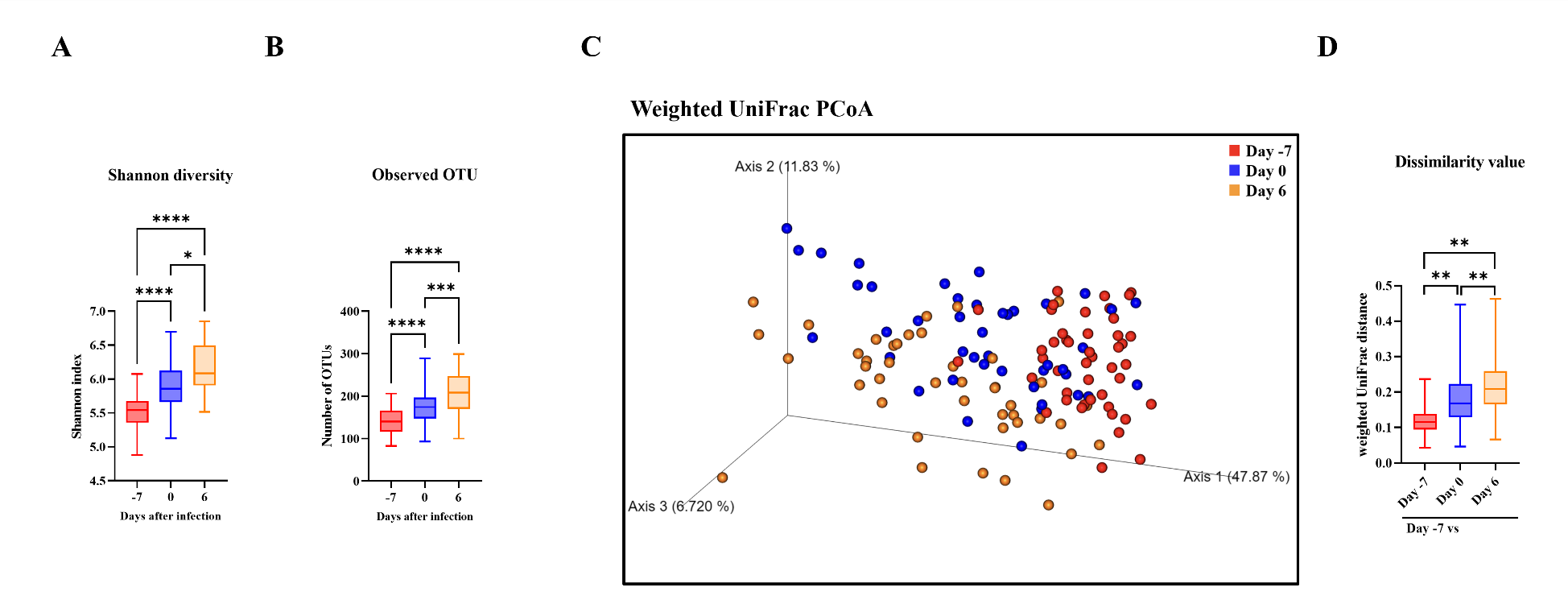


**Fig. S4** Changes in gut microbial community structure with influenza infection. (A) Shannon diversity indices and (B) observed OTU indices of the fecal microbiome at 10,885 sequences per sample (n = 43-45). Significance in (A) and (B) was determined using one-way ANOVA. (C) PCoA plot of the fecal microbiota structure based on weighted UniFrac metrics. (D) Pairwise weighted UniFrac distances of the day −7 group. PERMANOVA was applied (n = 45 permutations for the day −7 group; n = 43 permutations for the day 0 group; and n = 44 permutations for the day 6 group). Boxplots show the medians with interquartile ranges; whiskers indicate minima to maxima. Differences in microbial composition were evaluated using PERMANOVA based on weighted UniFrac distances. * *P* < 0.05, ** *P* < 0.01, *** *P* < 0.001, **** *P* < 0.0001


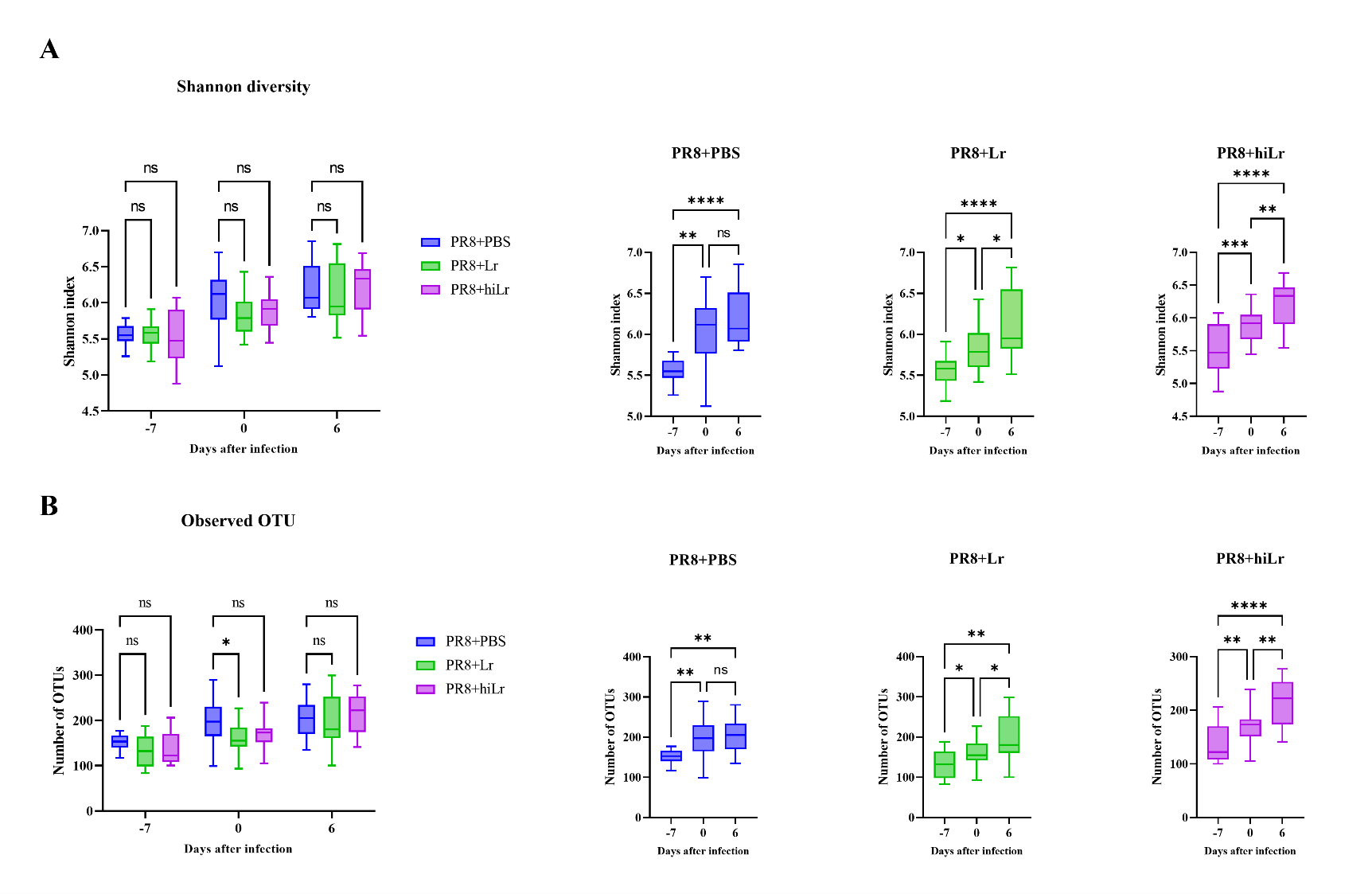


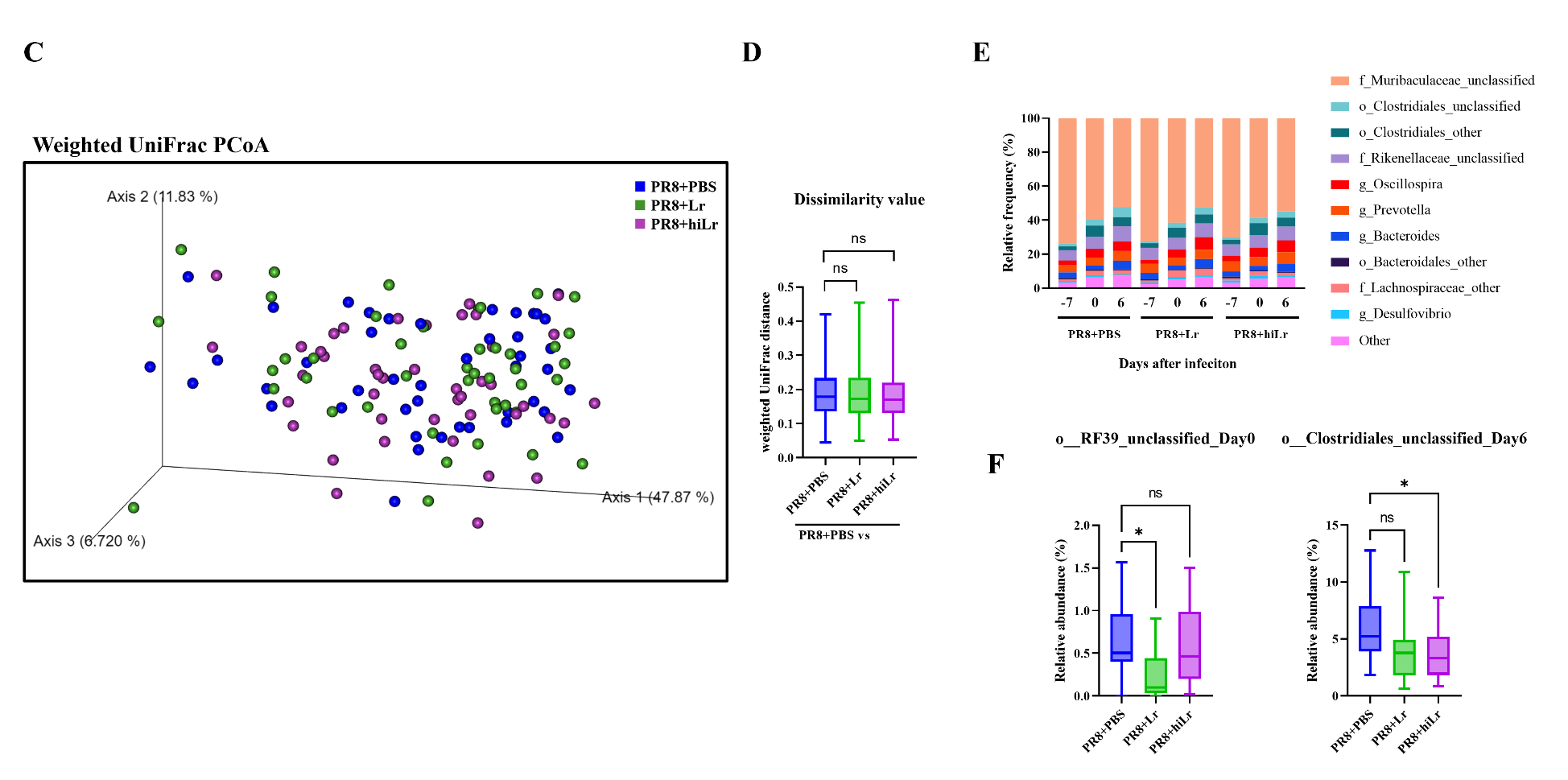


**Fig. S5** Changes in the gut microbial community structure in PR8-infected and *L. reuteri* KBL346-treated mice. (A) Shannon diversity and (B) observed OTU indices of the fecal microbiome at 10,885 sequences per sample (n = 13-15). Significance was determined using one-way ANOVA. (C) PCoA plot of the fecal microbiota structure based on weighted UniFrac metrics. (D) Pairwise weighted UniFrac distances to the control group. PERMANOVA was applied (n = 44 permutations for PR8 + PBS, n = 45 permutations for PR8 + live *L. reuteri* KBL346, n = 43 permutations for PR8 + heat-inactivated *L. reuteri* KBL346). (E) Genus-level average relative abundances. (F) Relative abundance of RF39_unclassified at day 0 and Clostridiales_unclassified at day 6. When appropriate, significance was determined using Welch and Brown–Forsythe ANOVA or the nonparametric Kruskal–Wallis test. Data in (A), (B), (D), and (F) are from one experiment and are the medians with interquartile ranges; whiskers extend from minima to maxima. * *P* < 0.05, ** *P* < 0.01, *** *P* < 0.001, **** *P* < 0.0001

**References**

1. Li J, Shiroyanagi Y, Lin G, Haqq C, Lin CS, Lue TF, Willingham E, Baskin LS (2006) Serum response factor, its cofactors, and epithelial–mesenchymal signaling in urinary bladder smooth muscle formation. Differentiation 74(1):30-39. https://doi.org/10.1111/j.1432-0436.2006.00057.x
2. Kwon H-K, Lee C-G, So J-S, Chae C-S, Hwang J-S, Sahoo A, Nam JH, Rhee JH, Hwang KC, Im SH (2010) Generation of regulatory dendritic cells and CD4+ Foxp3+ T cells by probiotics administration suppresses immune disorders. Proc Natl Acad Sci U S A 107(5):2159-2164. https://doi.org/10.1073/pnas.0904055107
3. Froy O, Chapnik N (2007) Circadian oscillation of innate immunity components in mouse small intestine. Mol Immunol 44(8):1954-1960. https://doi.org/10.1016/j.molimm.2006.09.026
4. Bukong TN, Maurice SB, Chahal B, Schaeffer DF, Winwood PJ (2016) Versican: a novel modulator of hepatic fibrosis. Lab Invest 96(3):361-374. https://doi.org/10.1038/labinvest.2015.152
5. Yoshihara-Hirata C, Yamashiro K, Yamamoto T, Aoyagi H, Ideguchi H, Kawamura M, Suzuki R, Ono M, Wake H, Nishibori M, Takashiba S (2018) Anti-HMGB1 neutralizing antibody attenuates periodontal inflammation and bone resorption in a murine periodontitis model. Infect Immun 86(5):10-1128. https://doi.org/10.1128/iai.00111-18
6. Donnelly DJ, Longbrake EE, Shawler TM, Kigerl KA, Lai W, Tovar CA, Ransohoff RM, Popovich PG (2011) Deficient CX3CR1 signaling promotes recovery after mouse spinal cord injury by limiting the recruitment and activation of Ly6C^lo^/iNOS^+^ macrophages. J Neurosci 31(27):9910-9922. https://doi.org/10.1523/JNEUROSCI.2114-11.2011
7. Zhao D, Alizadeh D, Zhang L, Liu W, Farrukh O, Manuel E, Diamond DJ, Badie B (2011) Carbon nanotubes enhance CpG uptake and potentiate antiglioma immunity. Clin Cancer Res 17(4):771-782. https://doi.org/10.1016/j.jneuroim.2008.12.006
8. Lemmers B, Salmena L, Bidere N, Su H, Matysiak-Zablocki E, Murakami K, Ohashi PS, Jurisicova A, Lenardo M, Hakem R, Hakem A (2007) Essential role for caspase-8 in Toll-like receptors and NFκB signaling. J Biol Chem 282(10):7416-7423. https://doi.org/10.1074/jbc.M606721200
